# Ruta Graveolens

**Published:** 1883-10

**Authors:** Ad. Lippe

**Affiliations:** Philadelphia


### RUTA GRAVEOLENS.

AD. LIPPE, M. D., PHILADELPHIA.

It is a well-confirmed observation that Ruta cures dimness of sight caused by overexertion of the eyes. First among the observations of the "provers" was "Symp. 38 (*Hahnemann's Mat. Med. Pura*): It is before the eyes, as when the sight is fatigued by reading too long." The intelligent healer who is not what malicious men call a symptom coverer, but who knows how to utilize our materia medica, has also cured many different affections of the eyes caused by overfatiguing the eye, from reading too much, from using them to excess in fine needlework, etc. We find Dr. Hughes, in the *British Journal of Homœopathy* for July, 1883, wind up his clinical lectures on Ruta by saying: "*Asthenopia is the morbid ocular condition here indicated as the sphere of Ruta!*" Bravo! here we have a generous contribution to the proposed Pathological Picture Book. Asthenopia sounds awfully "*scientific*," to be sure, but when the Greek roots are sifted it means only "*weakness of sight*." The unfortunate man who, so misled, gives to every patient suffering from asthenopia Ruta will become disgusted with such a caricature of the Homœopathic Healing Art as must result from using this Pathological Picture Book to guide him in applying the Law of the Similars. The true healer who knows how to use the materia medica will continue to cure *all* kinds of diseases of the eyes *caused by overexertion*, from the simple asthenopia all the way up to the fully developed cataract. He, the true healer, has a logical mind, and as a homœopathican he "individualizes;" he does not desire to be led into superannuated generalizations; he despises the Pathological Picture Book.

Ruta was known to the ancients. In the Code of Health of the School of Salerno—ninth century—we find the following under Ruta:

> Nobilis est Ruta quia lumina reddit acuta,
> Auxilio Rutæ, vir lippe videbis acute.
> Ruta viris minuit Venerem, mulieribus addit.
> Ruta facit castum, dat lumen et ingerit astum,
> Cocta jacit ruta de pulicibus loca tuta.*

Why then is it that scientific specialists, that the ever-increasing number of oculists, have forgotten the praises Hippocrates, the School of Salerno, Plinius, Dioscorides, Pythagoras, Boerhave, and

---

> * Of use to sight, a noble plant is Rue:
> O bleareyed man! 'twill sharpen sight for you!
> In man, it curbs love's strongest appetite;
> In women, it tends to amplify its might.
> Yet rue to chastity inclines mankind,
> Gives power to see and sharpens, too, the mind;
> And instantly, when in decoction, frees
> Your house forever from tormenting fleas.

others bestowed on the curative effects of Ruta in cases of weakness of vision? Why? because the indications for its use consisted in generalizations. What Hahnemann and his true followers have done by proving drugs on the healthy was that they were soon enabled to state with absolute certainty "*under what conditions* each separate drug would cure the sick." And now we must see professed homœopaths boldly demand the adoption of generalizations; demand a progress backward two thousand years! And just as two thousand years ago Ruta was highly praised as a great remedy to strengthen the sight, so comes out at present the great perverter of our school and tells his hearers, "*Asthenopia is the morbid ocular condition here indicated as the sphere of Ruta.*" Let him and his aider and abettor in the United States publish their Pathological Picture Book, let it be dedicated to "The School of Salerno," or, still better, to Hippocrates. Let them repudiate Hahnemann and his methods, seek recognition by the ancients, and form a new school on that basis.

